# Skin CD4^+^ memory T cells exhibit combined cluster-mediated retention and equilibration with the circulation

**DOI:** 10.1038/ncomms11514

**Published:** 2016-05-10

**Authors:** Nicholas Collins, Xiaodong Jiang, Ali Zaid, Bethany L. Macleod, Jane Li, Chang Ook Park, Ashraful Haque, Sammy Bedoui, William R. Heath, Scott N. Mueller, Thomas S. Kupper, Thomas Gebhardt, Francis R. Carbone

**Affiliations:** 1 Department of Microbiology and Immunology; Peter Doherty Institute for Infection and Immunity, The University of Melbourne, Melbourne, Victoria 3000, Australia; 2 Department of Dermatology and Harvard Skin Disease Research Center, Brigham and Women's Hospital, Harvard Medical School, Boston, Massachusetts 02115, USA; 3 Malaria Immunology Laboratory, QIMR Berghofer Medical Research Institute and Australian Infectious Diseases Research Centre, The University of Queensland, Queensland 4006, Australia

## Abstract

Although memory T cells within barrier tissues can persist as permanent residents, at least some exchange with blood. The extent to which this occurs is unclear. Here we show that memory CD4^+^ T cells in mouse skin are in equilibrium with the circulation at steady state. These cells are dispersed throughout the inter-follicular regions of the dermis and form clusters with antigen presenting cells around hair follicles. After infection or administration of a contact sensitizing agent, there is a sustained increase in skin CD4^+^ T-cell content, which is confined to the clusters, with a concomitant CCL5-dependent increase in CD4^+^ T-cell recruitment. Skin CCL5 is derived from CD11b^+^ cells and CD8^+^ T cells, with the elimination of the latter decreasing CD4^+^ T-cell numbers. These results reveal a complex pattern of tissue-retention and equilibration for CD4^+^ memory T cells in skin, which is altered by infection and inflammation history.

## Abstract (web summary)

Memory T cells are vital responders to skin inflammation, but cell localization and dynamics of exchange with the bloodstream are not clear. Here the authors use parabiosis and intravital microscopy to show that CD4^+^ memory T cells equilibrate with the circulation and cluster around hair follicles in response to CCL5-dependent responses to viral infection or contact sensitization.

## Graphical abstract

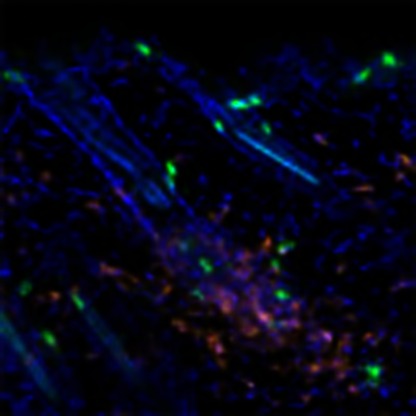

Many pathogens gain entry into the body via barrier surfaces such as the skin, gut and respiratory tract. Thus, effective immunity against these infections relies in part on T cells that access these body surfaces. Early studies in sheep showed that T cells constitutively recirculate between barrier tissues and the blood via the lymphatic system^1^. These T cells were found to be antigen experienced or memory cells^2^, meaning that memory T-cell recirculation through peripheral compartments contributes to specific immunity against infection. Blood-derived human T cells were subsequently found to partition into central (T_CM_) and effector (T_EM_) memory subsets, with the latter speculated to be the population involved in recirculating-surveillance of non-lymphoid organs^3^.

Missing from this rudimentary account of peripheral immunity was the possibility that at least some of the tissue T cells never returned to the blood. It is now clear that a proportion of memory cells are permanently lodged in non-lymphoid compartments^4^,^5^. These tissue-resident memory T (T_RM_) cells are best defined among the CD8^+^ subset, in which they seem to be distinct from circulating T_EM_ cells^6^. With this expanded understanding, it is clear that there is a complexity within the peripheral compartments, which can contain various mixtures of recirculating and resident memory populations^7^. Skin is one of the largest organs of the body and, at least in humans, is known to contain more memory T cells than are found in the circulation^8^. Although the T-cell composition in human skin is different to that found in mouse, some elements are common such as their preferential localization in the dermis and a predominance of CD4^+^ T cells over CD8^+^ T cells^7^,^8^,^9^,^10^.

Memory CD4^+^ T cells can protect peripheral tissues such as the skin and reproductive tract against infection with pathogens such as herpes simplex virus (HSV)^10^,^11^,^12^. A common feature of many tissue T cells is their accumulation in clusters, often including professional antigen presenting cells (APCs), such as macrophages and dendritic cells^12^,^13^. These aggregates are important in CD4^+^ T-cell residency in the female reproductive tract (FRT)^12^ as well as CD8^+^ T-cell retention in the intestine^13^, suggesting that they may represent a more general mechanism of T cell accumulation in the periphery. In particular, T-cell clusters have been observed in human and mouse skin, especially around appendages such as hair follicles^8^,^9^,^10^. Separately, evidence for preferential exit of CD4^+^ T cells from the skin exists for various species including sheep, mouse and humans^2^,^10^,^14^ suggesting that these T cells make up the main recirculating population. Here, we show that the skin CD4^+^ T cells persist in peri-follicular clusters, with the majority in equilibrium with the blood during steady-state. Infection results in a prolonged increase in skin chemokine production and a concomitant increase in T-cell recruitment from the blood. This argues for a dynamic CD4^+^ T-cell compartment in the skin, with an equilibrium set-point that is altered by a history of infection and inflammation.

## Results

### Memory CD4^+^ T cells recirculate between naive skin and blood

**Figure 1:**
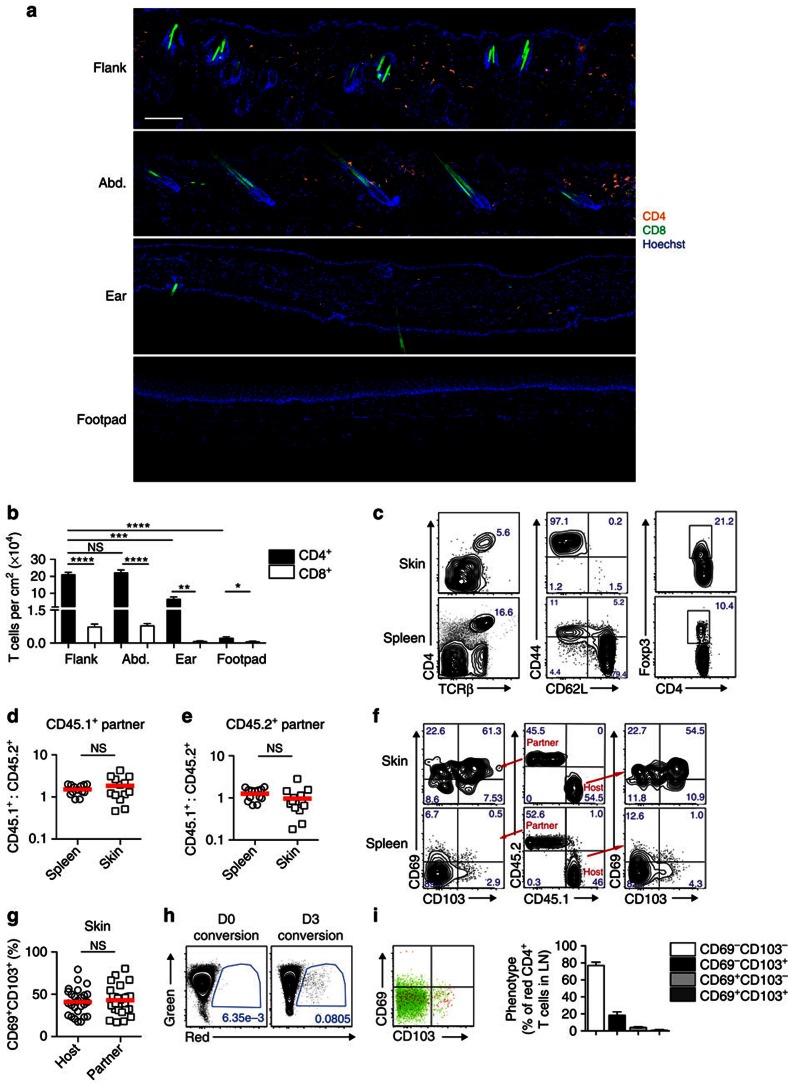
Memory CD4^+^ T cells recirculate between naive skin and blood. (**a**) Microscopy of naive mouse flank, abdomen, ear and footpad skin, detecting CD4 (orange) and CD8 (green) using fluorescently labelled antibodies. Cell nuclei (blue) are visualized by Hoechst 33342. Scale bar, 100 μm. (**b**) Number of T cells in sections of naive flank, abdomen, ear and footpad skin; *n*=7–14. (**c**) Phenotype of CD4 T cells in naive flank skin. Left plots gated on CD45.2^+^ live cells, middle and right plots gated on TCRβ^+^ CD4^+^ cells. (**d**,**e**) Naive CD45.1^+^ and CD45.2^+^ mice were surgically joined for 12–16 weeks then assessed by flow cytometry. Graphs display the ratio of CD45.1^+^: CD45.2^+^ CD4 T cells in the spleen and skin of the CD45.1^+^ (**d**) and CD45.2^+^ (**e**) partners. (**f**,**g**) CD69 and CD103 expression by host and partner CD4 T cells from spleen and skin of parabiotic mice in **d**,**e**; middle plots gated on CD4^+^ cells from the CD45.1^+^ partner. (**h**) Kaede red CD4 T cells in the brachial lymph node analysed immediately after and 3 days following photo-conversion. (**i**) CD69 and CD103 expression of kaede red and green CD4 T cells in the brachial lymph node 3 days following photo-conversion. Each symbol in **d**,**e**,**g** represents an individual mouse; *n*=26 mice from 13 pairs. NS, not significant; *P<0.05, **P<0.01, ***P<0.001, **** *P*<0.0001; two-tailed paired *t*-test. Data in **a**,**c**,**f**,**h**,**i**) are representative of at least two experiments. Data in **b**,**d**,**e**,**g** are pooled from two to three experiments. Mean (**d**,**e**,**g**), mean and s.e.m. (**b**,**i**). Numbers in plots of **c**,**f** represent frequency of events in the respective gates. Abd; abdominal skin; LN, lymph node.

Since human skin intrinsically contains large numbers of T cells, we wanted to determine whether this was the case in naive mouse skin that had not been purposely infected. We examined skin from four areas, namely the ear, flank, abdomen and footpad. Histological approaches were used to determine the absolute number of T cells in skin, as tissue processing for flow cytometry can significantly underestimate the numerical content^15^. Flank and abdominal skin had the highest T-cell density (Fig. 1a,b), with ear and footpad containing fewer to virtually no T cells, respectively. Given the high T-cell content in the mouse flank, we focused our attention on this region. Flank skin T cells were predominantly CD4-positive and were generally located within the dermis (Fig. 1a). These CD4^+^ T cells lacked CD62L and often Foxp3, but expressed both CD44 and TCRβ (Fig. 1c). Thus, the majority of the CD4^+^ cells in resting flank skin are classified as conventional memory T cells.

Mouse flank contained ∼0.2 × 10^6^ CD4^+^ T cells per cm^2^ (Fig. 1b). A 20 g mouse has ∼36 cm^2^ of skin surface area^16^, meaning that there were a total of 7.2 × 10^6^ memory CD4^+^ T cells in mouse skin. By comparison, there are roughly 30 × 10^6^ CD4^+^ T cells in the secondary lymphoid organs^17^. In our cohort, ∼15% of these expressed CD44 (Fig. 1c), which equates to ∼4.5 × 10^6^ memory T cells in these lymphoid compartments. Thus, to a rough approximation, CD4^+^ memory T cells were evenly distributed between secondary lymphoid tissues and mouse skin. In sharp contrast to the CD4^+^ T cells, resting skin contained at least tenfold fewer CD8^+^ T cells (Fig. 1b), found predominantly in the epithelial compartment (Fig. 1a).

Given the high proportion of CD4^+^ memory T cells in resting skin, we wanted to determine whether these were confined to this compartment or recirculated between tissue and lymphoid organs via the blood. To this end, we surgically linked the blood supply of pairs of naive mice expressing either CD45.1 or CD45.2. Figure 1d,e and Supplementary Fig. 1A–B show the equilibration of both splenic and skin CD4 T-cell populations by 12–16 weeks after parabiosis, with nearly equal proportions of cells bearing the respective markers in both mice of each pair. Thus, it appeared that CD4^+^ T cells found in mouse skin were in equilibrium with the circulation. Surprisingly, although CD69 and CD103 are usually associated with tissue residency^6^, almost half of the CD4^+^ T cells that entered the skin via the blood, defined as CD45.2^+^ T cells in the CD45.1^+^ parabiont, expressed these surface molecules, which was similar to the host-derived cells (Fig. 1f,g). Importantly, the splenic T cells were uniformly negative for both these markers (Fig. 1f). To investigate the phenotype of CD4^+^ T cells that had egressed the skin, kaede mice expressing a photo-convertible protein that changes from green to red upon exposure to violet light^18^,^19^ were used. Flank skin was photo-converted and red cells that had left the skin and entered the draining lymph node were examined 3 days later (Fig. 1h). In terms of CD69 and CD103 expression, red-converted kaede CD4^+^ T cells were either double negative or only expressed CD103 (Fig. 1i). Since the majority of photo-converted kaede cells expressed CD69 and/or CD103 in the skin (Fig. 1f), this argued that these molecules are likely modulated as CD4^+^ T cells enter and leave the skin.

### Half of all skin CD4^+^ T cells cluster around hair follicles

**Figure 2:**
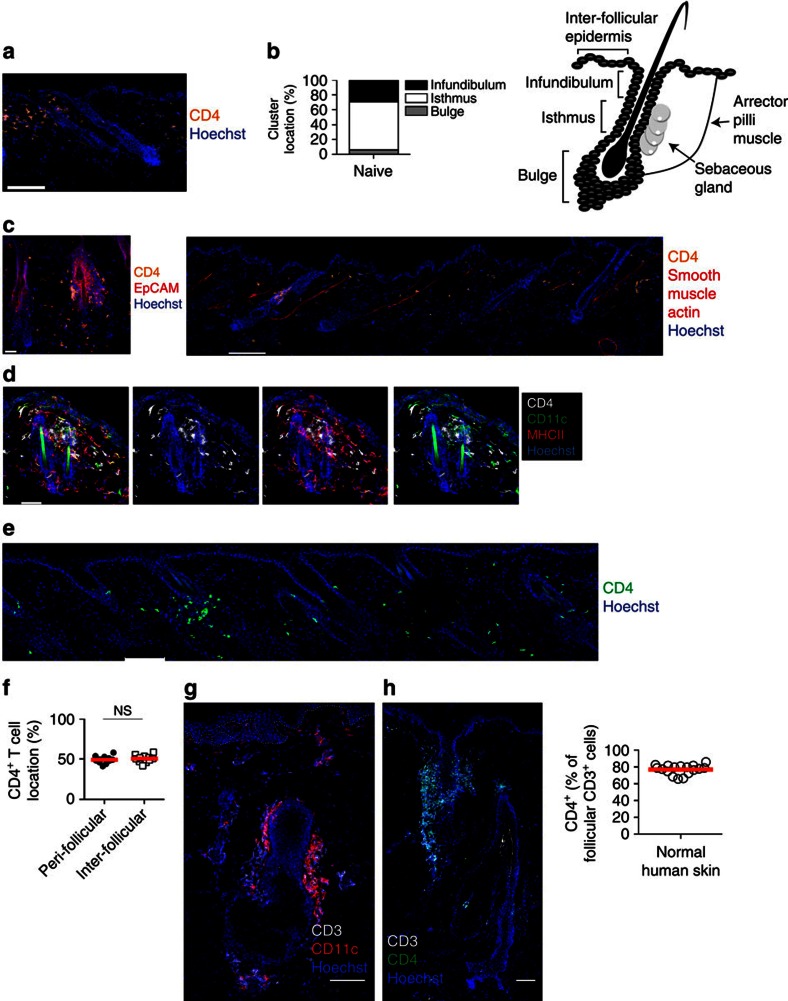
CD4 T cells and APC cluster around hair follicles at steady state. (**a**) Naive flank skin, detecting peri-follicular CD4 T cells (orange). Scale bar, 100 μm. (**b**) Location of peri-follicular clusters; *n*=10, determined by (**c**) stains that recognize EpCAM (left image) or the arrector pilli muscle (identified by anti-smooth muscle actin) (right image). Scale bar, 30 and 100 μm, respectively. (**d**) Peri-follicular cluster, detecting CD4 (white), CD11c (green) and MHC class II (red) positive cells. Scale bar, 50 μm. (**e**) Naive skin of MHC II^−/−^ mice injected intradermally with *in vitro* activated wild-type GFP^+^ CD4 T cells (green). Scale bar, 100 μm. (**f**) Location of CD4 T cells within the dermis of naive skin; *n*=11. (**g**,**h**) Microscopy of healthy human skin staining for (**g**) CD3 (white) and CD11c (red) and (**h**) CD3 (white) and CD4 (green). Scale bar: 100 μm (**g**); and 50 μm (**h**). Graph in **h** shows the frequency of peri-follicular CD3^+^ cells that are CD4^+^, *n*=17. Each symbol in **f** represents the indicated CD4 T-cell population in a single mouse or skin sample from an individual person (graph of **h**). NS, not significant (two-tailed paired *t*-test). Data are representative of four (**a**) or two (**c**–**e**) experiments with two to five mice, or pooled from two (**b**) or three (**f**) experiments. Graph in (**h**) is pooled from 16 individual experiments. Line in **f**,**h** indicates mean.

Human skin T cells are known to accumulate around appendages such as hair follicles and experimentally, lymphocyte clusters are important for T-cell retention in the gut and FRT after infection^8^,^12^,^13^. We found clusters in naive mouse flank skin, with the majority appearing as peri-follicular (Fig. 2a). The clusters showed biased accumulation around the isthmus region (Fig. 2b). The keratinocytes in this site express EpCAM at high levels^20^, with staining localized just above the bulge; the latter conveniently identified by the insertion point of the arrector pili muscle (Fig. 2c). The CD4^+^ T-cell clusters also contained major histocompatibility complex (MHC)-class II^+^ cells (Fig. 2d), roughly half of which were dendritic cells (CD11c^+^) and the other half were CD11c^−^ and likely to be macrophages, staining positive for CD64 and F4/80 (Fig. 2d and Supplementary Fig. 2). It should be noted that not all hair follicles had discernable peri-follicular clusters, but the T-cell clusters always contained MHC class II^+^ cells. Peri-follicular T-cell accumulation did not appear to be antigen dependent, since activated CD4^+^ T cells injected into skin of MHC class II-deficient mice could be found around follicles (Fig. 2e). In addition, nearly half of all dermal CD4^+^ T cells were found in close association with hair follicles, with the rest dispersed over the inter-follicular dermis (Fig. 2f).

Healthy human skin was next examined to see if it showed similar patterns of T-cell accumulation. As with mouse skin, CD3^+^ and CD11c^+^ cells were found in peri-follicular clusters that were predominantly localized to the isthmus (Fig. 2g) and the majority of follicular CD3^+^ cells were CD4^+^ (Fig. 2h). Overall, the skin CD4^+^ T cells were broadly distributed between APC-containing peri-follicular clusters and the inter-follicular dermis.

### Infection increases skin CD4^+^ T-cell recruitment and retention

**Figure 3:**
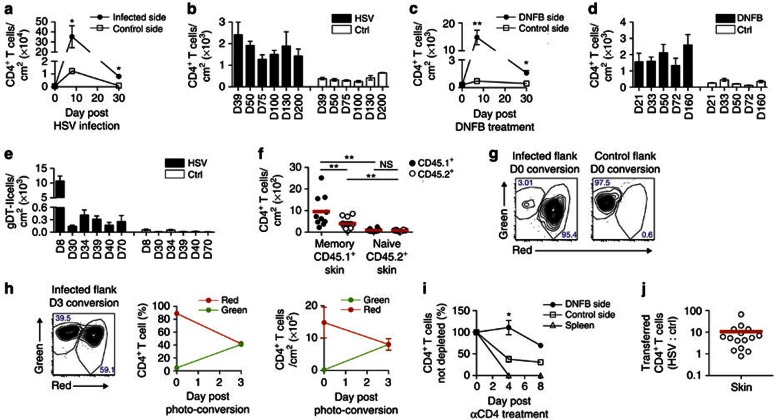
Infection increases skin CD4^+^ T-cell recruitment and retention. (**a**) CD4 T cells in HSV-infected skin over time until day 30 and (**b**) at memory; *n*=3–5 mice. (**c**) CD4 T cells in DNFB-treated skin over time until day 30 (**d**) and at memory; *n*=5 mice. (**e**) 10^4^ gDT-II cells were transferred into naive B6 mice, which were infected the following day. GDT-II cells in HSV-infected skin from day 8–70; *n*=3–5 mice. (**f**) CD45.1^+^ mice were treated with DNFB 3 weeks before being joined to naive CD45.2^+^ mice for 8 weeks. Enumeration of CD45.1^+^ and CD45.2^+^ CD4 T cells in skin; *n*=10 pairs. (**g**,**h**) Skin of HSV-infected kaede mice (days 30–50) was photo-converted. Proportion of red/green CD4^+^ T cells (**g**) immediately or 3 (**h**) days following photo-conversion. (**h**) Frequency and number of red/green CD4 T cells in previously HSV-infected skin, *n*=5–15. (**i**) DNFB memory (days 28–70) mice treated with an anti-CD4 antibody. Average percentage of CD4 T cells compared with PBS-treated mice; *n*=3–5 mice at each time point. (**j**) Splenic CD4 T cells from CD45.2^+^ HSV-1 memory mice (days 33–63) were transferred into infection-matched CD45.1^+^ mice. Transferred CD4 T cells on the infected side relative to control 7–8 days after transfer; *n*=15. Each symbol represents an individual mouse. NS, not significant; *P<0.05, **P<0.01 two-tailed paired *t*-test (**a**,**c**,**i**) or two-tailed Wilcoxon matched-pairs signed rank test (**f**). Data representative of at least two experiments (**a**,**c**,**h**) or one for each time point (**b**,**d**). In **e**, day 8 is pooled from two experiments and each memory time point of one. Data pooled from two experiments (**f**): four (day 4); two (day 8) in **g**; and five in **j**. Flow cytometry plots (**g**,**h**) representative of at least two similar experiments. Mean (**f**,**j**) and mean and s.e.m. (**a**–**e**,**h**,**i**). Numbers in flow cytometry plots represent frequency. Ctrl; uninfected flank from HSV-1-infected/DNFB-treated mice.

It has been reported that HSV infection results in a sustained increase in the number of CD4^+^ T cells found in the FRT^12^. This was also true of the skin after infection with this virus (Fig. 3a,b) and treatment with the contact sensitizing agent 1-Fluoro-2,4-dinitrobenzene (DNFB) (Fig. 3c,d), as determined by flow cytometric analysis of digested skin. HSV resulted in extensive T-cell infiltration of CD4^+^ T cells at the site of infection, with most being lost by day 30 after inoculation (Fig. 3a). After that time, there was a stabilization of CD4^+^ T-cell numbers at levels elevated from those found in untreated skin of the same animals (Fig. 3b). A similar pattern was also seen after treatment of skin with DNFB (Fig. 3c,d).

On average, there were sixfold more T cells persisting long term after infection compared with either non-infected regions from the same mouse or resting skin from animals not subjected to infection (Fig. 3a,b). A similar increase was seen following DNFB treatment, with the difference between treated and non-treated flanks being around eightfold (Fig. 3d). Short-pulse (5 min) *in vivo* administration of PE-labelled anti-CD3 antibody showed that greater than 95% of skin T cells left after infection were not stained (Supplementary Fig. 3), meaning that they were largely contained within the tissue parenchyma. Finally, HSV-inoculated skin not only had more total CD4^+^ T cells as shown in Fig. 3a,b but also had more virus-specific cells; the latter enumerated using transgenic T cells (gDT-II) specific for the class II-restricted determinant from the HSV gD protein^21^ (Fig. 3e).

To better understand the contribution of T-cell retention versus recirculation after skin challenge, we performed parabiosis between DNFB-treated and non-treated animals. Mice were joined in these experiments, where one (CD45.1) had resolved DNFB treatment while the second (CD45.2) was untreated. At analysis, splenic T cells were in equilibrium, with ratios of CD45.1^+^ to CD45.2^+^ cells of roughly 1 to 1 (Supplementary Fig. 4). In skin, there were dramatically more CD4^+^ T cells in the treated CD45.1^+^ partner (Fig. 3f). Strikingly, there was an increase in both host (CD45.1) and recruited (CD45.2) cells (Fig. 3f). This meant that DNFB-treatment increased CD4 T-cell migration into the tissue well after application and lesion resolution. In addition, there were roughly twice as many host (CD45.1) T cells in the DNFB-treated flanks as there were recruited (CD45.2) T cells (Fig. 3f), pointing to enhanced retention or residency for the host T cells. Similar results were obtained when both partners had resolved HSV infection before surgery. In this case, more host-derived cells were also observed in previously infected skin, whereas similar numbers of host and partner CD4^+^ T cells were found in uninfected areas from the same mice (Supplementary Fig. 5A). Furthermore, a higher number of host-derived cells were present in previously infected skin compared with uninfected areas (Supplementary Fig. 5A). Thus, while CD4^+^ T cells in resting skin were largely recirculating, in previously inflamed skin there appeared to be heterogeneity, with both recirculating and resident populations present.

Kaede mice were next used to investigate the contribution of resident and recirculating cells to the memory CD4^+^ T-cell population. Skin that had resolved HSV infection was photo-converted, resulting in ∼90% of CD4^+^ T cells expressing the red form of the kaede protein (Fig. 3g). Control flank skin of the same mice was left unconverted, demonstrating that almost all of the cells at this site remained green (Fig. 3g). In previously infected skin, the proportion and number of red cells decreased after 3 days and there was a concomitant increase in those that were green, so that approximately half of each population was present (Fig. 3h). This suggests that roughly half the memory CD4^+^ T cells in skin are recirculating (green), while the remaining cells are retained or resident (red), consistent with there being heterogeneity within this population.

Administration of T-cell-depleting antibodies results in the elimination of skin T cells that re-enter the circulation, while sparing those that remain in the tissue^7^,^22^. Increased retention by sensitized skin was further demonstrated by examining the loss of skin T cells after elimination of all CD4^+^ cells from the circulation. Intravenous injection of a depleting anti-CD4 antibody rapidly eliminated T cells from the lymphoid organs from DNFB-treated animals (Fig. 3i). Cells were also gradually lost in the skin, most likely as a consequence of their egress into the circulation, but at a slower rate than seen in spleen. This loss was more rapid in unsensitized skin compared with the previously inflamed regions from the same animals (Fig. 3i and Supplementary Fig. 6A), consistent with an increased retention of the T cells. Finally, enhanced T-cell recruitment was demonstrated for HSV-infected skin. This was done by isolating memory CD4^+^ T cells from previously infected mice and then reinfusing them into animals that had resolved the same type of infection. As shown in Fig. 3j and Supplementary Fig. 6B, there was preferential accumulation of the memory cells in skin that had been subjected to prior infection relative to uninfected skin on the opposite flank. Combined, the data in Fig. 3 argue that sensitization and infection resulted in enhanced recruitment of memory cells from the circulation into treated skin, in addition to the generation of a long-term resident population.

### Skin CD4^+^ T-cell retention involves CCL5-dependent clustering

**Figure 4:**
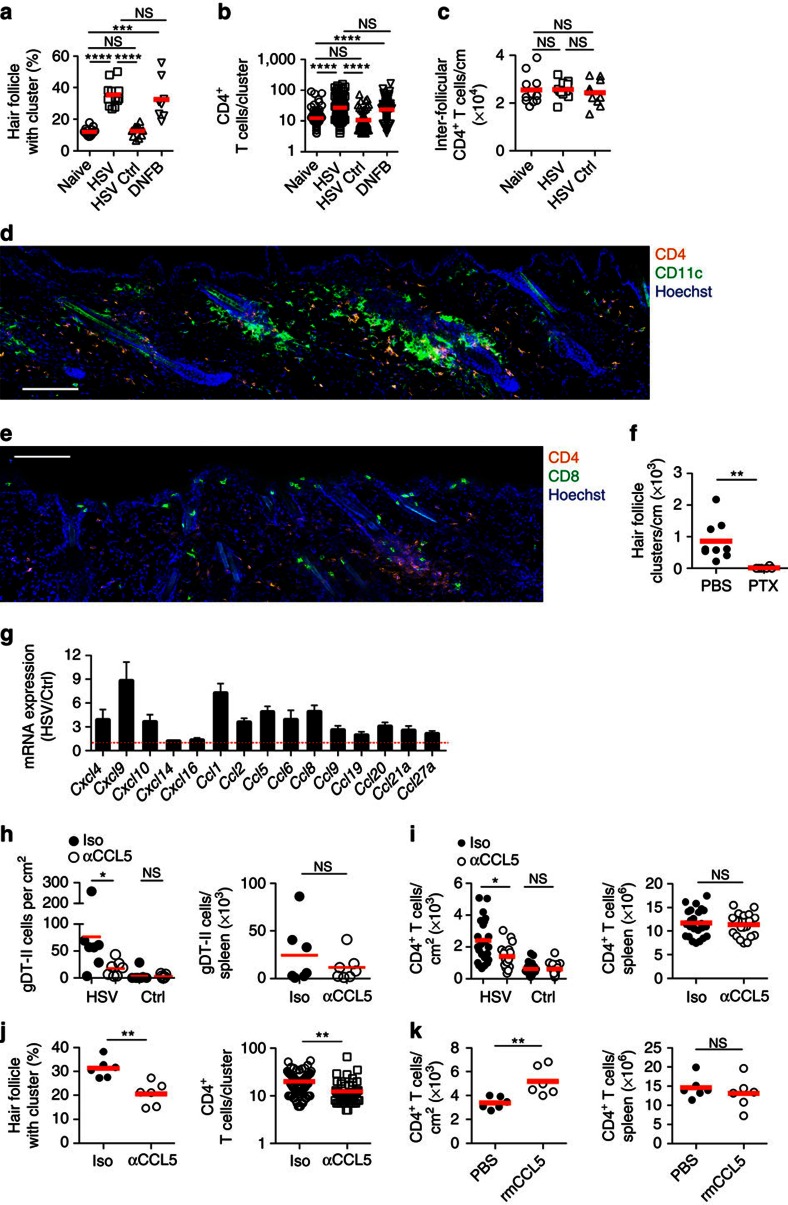
Hair follicle clusters account for the increase in skin CD4 T cells. (**a**) Hair follicle cluster frequency; *n*=8–15 and (**b**) number of CD4 T cells per cluster; *n*=107–167 clusters from seven to eight mice. (**c**) Number of inter-follicular dermal CD4 T cells; *n*=10–12. (**d**,**e**) HSV memory skin detecting (**d**) CD4 (orange) and CD11c (green) and (**e**) CD4 (orange) and CD8 (green). Scale bar, 100 μm. (**f**) Number of peri-follicular clusters incorporating pertussis toxin treated *in vitro* activated CD4 T cells, 1 week after intradermal injection into the skin of naive mice; *n*=6–9. (**g**) Chemokine mRNA transcripts in HSV memory skin relative to control skin (days 30–40); *n*=8. (**h**,**i**) HSV memory mice treated with an anti-CCL5 antibody before analysis of (**h**) gDT-II cells and (**i**) total CD4 T cells; *n*=7 and 20–24, respectively. (**j**) Hair follicle cluster frequency and number of CD4 T cells per cluster on the infected side of anti-CCL5-treated HSV memory mice; *n*=6 mice or 61–82 clusters pooled from six mice, respectively. (**k**) Enumeration of CD4^+^ T cells following intradermal injection of recombinant mouse CCL5. Symbols in (**a**,**c**,**f**,**h**,**i**; left graph of **j** and **k**) represent a single mouse. In (**b** and the right graph of (**j**) symbols represent the number of CD4^+^ T cells in a single peri-follicular cluster pooled from seven to eight and six mice respectively, from two experiments. NS, not significant; * *P*<0.05, ** *P*<0.01, *** *P*<0.001, **** *P*<0.0001 (two-tailed unpaired *t*-test (right of **i**, left of **j** and **k**) or two-tailed Mann–Whitney test (**a**–**c**,**f**,**h**; right of **i**; and left of **j**,**k**). Images (**d**,**e**) representative of four experiments with two to four mice. Data pooled from two to four (**a**,**c**,**f**), two (**h**,**j**,**k**) or seven (**i**) experiments with two to five mice. Mean (**a**–**c**,**f**,**h**–**k**), mean and s.e.m. (**g**). Iso; isotype control antibody; PTX, Pertussis toxin, rm; recombinant mouse.

Histological examination showed that the increase in skin CD4^+^ T cells persisting at memory post infection or DNFB sensitization resulted from an increase in the proportion of hair follicle-associated clusters (Fig. 4a) combined with an increase in the average number of T cells within a given cluster (Fig. 4b). Importantly, there was little change in the number of T cells in the inter-follicular dermis (Fig. 4c), meaning that T cell increases were almost completely confined to the clusters. These hair follicle clusters contained a high density of cells that expressed CD11c (Fig. 4d), as well as some CD8^+^ T cells (Fig. 4e).

To examine the role of chemokines in cluster formation, we began by showing that activated T cells injected into the dermis aggregated around the hair follicles (Fig. 4f). Blanket inhibition of chemokine signalling, by treating T cells with pertussis toxin (Ptx)^23^ before intradermal injection, dramatically altered the pattern of T-cell distribution by abolishing T-cell aggregation around the hair follicles (Fig. 4f). Examination of chemokine expression at memory showed that infection resulted in sustained upregulation of transcripts encoding for a variety of different chemokines, including CCL1, CCL2, CCL5, CCL8, CXCL9 and CXCL10 compared with control flank skin from the same animals (Fig. 4g). The chemokines CCL1, CCL2 and CCL8 did not appear to play an essential role in the enhanced recruitment or retention of CD4^+^ T cells in HSV-infected skin at memory, with no decrease in cell number observed in infected or control flanks of CCR8 (CCL1 and CCL8 receptor) or CCR2 (CCL2 receptor) deficient mice (Supplementary Fig. 7A–B). Furthermore, a significant decrease in number was not observed in infected or control skin following treatment of mice with the CXCR3 inhibitor AMG487 (Supplementary Fig. 7C), suggesting that CXCL9 and CXCL10 also do not play an appreciable role.

It had previously been shown that CCL5 was important for CD4^+^ T-cell clustering and tissue retention after HSV infection of the FRT^12^. We found that this chemokine also played a role in HSV infection of the skin with fewer gDT-II cells (Fig. 4h) and total CD4^+^ T cells (Fig. 4i) left in skin at memory after 4 days of treatment with an anti-CCL5 antibody, whereas the number in non-infected flank or in the spleen was unaffected (Fig. 4h,i). A decrease in CD4^+^ T-cell number following anti-CCL5 treatment resulted in a corresponding reduction in the proportion of hair follicles with associated clusters and the average number of CD4^+^ T cells in clusters in skin subjected to HSV infection (Fig. 4j). In addition, intradermal injection of recombinant mouse CCL5 into naive flank skin resulted in a local increase in number of CD4^+^ T cells at that site (Fig. 4k), indicating that this chemokine alone was sufficient to mediate enhanced recruitment and retention in skin. Overall, these data argue that CD4^+^ T-cell retention in skin is largely confined to the peri-follicular clusters and is in part dependent on the chemokine CCL5.

### CD8^+^ T-cell-produced CCL5 increases skin CD4^+^ T-cell content

**Figure 5:**
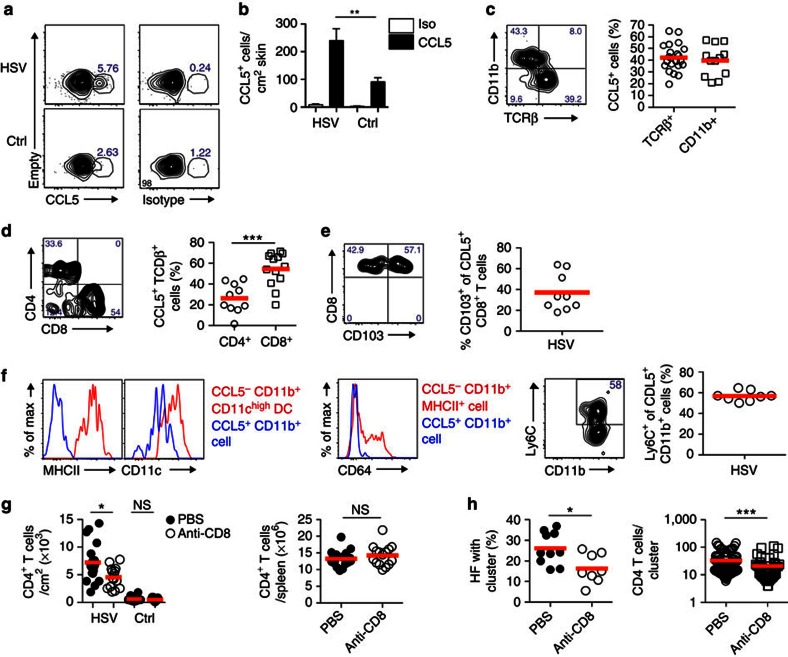
CD8^+^ T-cell-produced CCL5 increases skin CD4^+^ T-cell content. (**a**) Flow cytometry plots staining for CCL5 in previously HSV-infected or control skin at memory (days 40–100), gated on live CD45.2^+^ cells. Representative of three experiments with three to five mice. (**b**) Number of CCL5^+^ cells in previously HSV-infected and control skin (days 40–100), determined by *in situ* intracellular cytokine staining. (**c**) TCRβ and CD11b expression of CCL5^+^ cells in previously HSV-infected skin. (**d**) CD4 and CD8 expression of CCL5^+^ TCRβ^+^ cells. (**e**) CD103 expression of CCL5^+^ TCRβ^+^ CD8^+^ cells. (**f**) MHC class II and CD11c expression of CCL5^+^ CD11b^+^ cells (blue) compared with CCL5^−^ CD11b^+^ CD11c^high^ DC (red) from the same sample. CD64 expression of CCL5^+^ CD11b^+^ cells (blue) is also shown compared with CCL5^−^ CD11b^+^ MHC II^+^ cells (red) in the same sample. Flow cytometry plot shows Ly6C and CD11b expression by CCL5^+^ CD11b^+^ cells. Representative of three experiments with three to five mice. (**g**,**h**) Mice were depleted of CD8^+^ T cells before HSV infection. Graphs show number of CD4^+^ T cells in skin and spleen at these times (**g**), as well as follicle cluster frequency and number of CD4 T cells per cluster in previously infected skin (**h**). Symbols in **c**,**d**,**e**,**g** and left graph of **h** represent a single mouse. In the right graph of **h**, symbols represent the number of CD4^+^ T cells in a single peri-follicular cluster pooled from 8 to 10 mice (55–87 clusters), from two experiments. NS, not significant; *P<0.05, **P<0.01, ***P<0.001, two-tailed Mann–Whitney test). Data pooled from three to six experiments (**b**–**f**,**g**) with two to five mice per group or two experiments with four to five per group (**h**). Mean (**c**–**e**,**f**–**h**), mean and s.e.m. (**b**) Numbers in flow cytometry plots represent frequency.

The cellular source of CCL5 in previously HSV-infected skin was identified using an *ex vivo* intracellular cytokine staining assay^24^. This revealed that there were more CCL5^+^ cells in infected skin compared with control areas in terms of both frequency and number (Fig. 5a,b), consistent with the elevated mRNA transcripts in Fig. 4g. The majority of CCL5 was produced by cells that were either TCRβ^+^ or CD11b^+^ (Fig. 5c). Of the T cells, most were CD8^+^ (Fig. 5d) with heterogeneous expression of CD103 (Fig. 5e), while a considerably smaller population of CD4^+^ cells also produced CCL5 (Fig. 5d). The CD11b^+^ cells lacked expression of MHC II and had intermediate CD11c levels compared with CD11c^high^ DC (Fig. 5f). These cells also had low levels of CD64 and more than half expressed Ly6C (Fig. 5f), with this phenotype most closely resembling a population of monocyte-derived DC in skin at steady state^25^. Thus, CCL5 appears to be produced by different cells within the hair follicle clusters.

Given that CD8^+^ T cells were localized in peri-follicular clusters (Fig. 4e) and produced CCL5 (Fig. 5d), we next investigated if their absence would result in a reduction of memory CD4^+^ T cells in skin. To address this, mice were treated with an anti-CD8 antibody before HSV infection, then every 5 days until the time of analysis 1 month later. This approach resulted in a drastic reduction of CD8^+^ T cells in the skin and spleen at memory (Supplementary Fig. 8A), but it did not affect the ability of CD4^+^ T cells to infiltrate the skin during the acute response at day 8 (Supplementary Fig. 8B). Importantly, a significant reduction in number of CD4^+^ T cells was observed in previously infected skin at memory, while the number on the control flank and spleen remained the same (Fig. 5g). This decrease mirrored a decrease in frequency of follicle clusters and number of CD4^+^ T cells per cluster (Fig. 5h). The reduction appeared to be confined to the CD4^+^ T cells, since the number of MHC II^+^ APCs was not altered (Supplementary Fig. 8C). Altogether these results suggest that CD8^+^ T-cell-derived CCL5 plays a significant role in maintaining elevated numbers of CD4^+^ T cells in the skin long term following an infection.

### Peri-follicular CD4^+^ T cells exhibit high migration velocity

**Figure 6:**
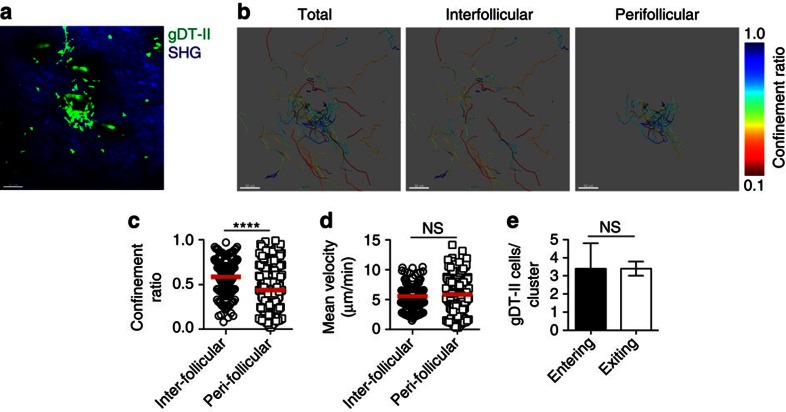
Peri-follicular CD4^+^ T cells exhibit high migration velocity. (**a**) Maximum intensity projection of clustered gDT-II cells (green) around a hair follicle (auto-fluorescent hair in green) at memory (30–70 days) post infection with HSV-1. The dermis is identifiable by collagen (second harmonic generation, blue). Scale bar, 50 μm. (**b**) Cell migration tracks from **a**, colours represent confinement ratio, with blue cells being the most confined and red the least. (**c**) Average confinement ratio and (**d**) mean velocity of gDT-II cells in the dermal inter-follicular space or peri-follicular clusters at memory following HSV infection. (**e**) The number of gDT-II cells that enter and exit hair follicle clusters. Symbols in **d**,**e** represent individual gDT-II cells. NS, not significant; ****P<0.0001 (two-tailed Mann–Whitney test (**c**–**e**)). Data is pooled from five movies from five mice (**c**–**e**). Mean (**c**,**d**), mean and s.e.m. (**e**).

Given that the data showed that the clusters were associated with tissue retention in combination with increased recruitment, we wanted to compare the migration characteristics of T cells in the peri- and inter-follicular compartments. Examination of the HSV-specific gDT-II cells between and around the hair follicles by two-photon microscopy after virus infection showed that CD4^+^ T cells in both locations were motile and migrating rapidly (Supplementary Video 1). T cells clustered immediately adjacent to hair follicles, either swarming to one side or they completely migrated around the follicular shaft. To calculate T-cell dynamics around or between the clusters, we defined cluster cells as those gDT-II T cells remaining for more than 10 min within 50 μm from the cluster centre, with that distance being the average cluster diameter. The remainder were attributed as being non-cluster or inter-follicular in nature. Tracks of cluster and non-cluster T cells are shown in Fig. 6a,b and Supplementary Video 2 for one such analysis. The cluster-associated T cells had a significantly higher confinement ratio than the inter-follicular cells (Fig. 6c), consistent with the former T cells being restricted in their pattern of migration around a focal region. However despite these constraints, T-cell migration velocity was largely the same for cluster and non-cluster cells (Fig. 6d). Finally, it was found that a proportion of T cells were able to either enter or exit cluster regions during the period of observation, indicative of flux in the composition of the clusters (Fig. 6e). Thus, it appeared that while peri-follicular cluster-associated CD4^+^ T cells were spatially constrained, they exhibited highly dynamic movement, with migration velocities similar to cells found elsewhere in the dermis.

### IFNγ-producing skin CD4^+^ T cells localize to hair follicles

**Figure 7:**
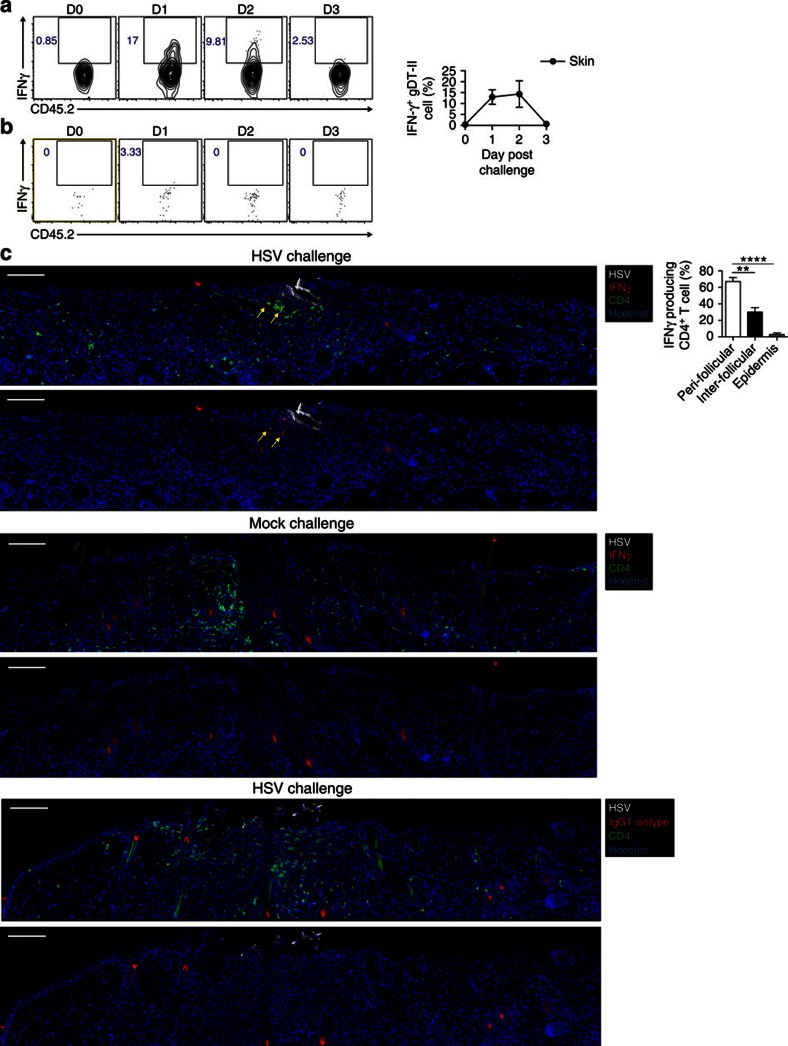
IFNγ-producing skin CD4^+^ T cells localize to hair follicles. (**a**,**b**) 10^4^ gDT-II cells were transferred to μMT mice the day before HSV infection, then challenged with this virus at least 30 days later. Flow cytometry plots and graph show the frequency of gDT-II cells producing IFNγ in the skin (**a**) and draining lymph node (**b**) at days 0–3 following challenge, *n*=2–7. (**c**) Images of previously HSV-infected μMT mice challenged at days 28–34 with HSV (top and bottom) or mock (middle) 30 h prior. Skin sections stained with HSV (white), CD4 (green) and IFNγ (red) in top and middle images, or an isotype control (red) for the bottom. All images are shown with and without the CD4 stain. Yellow arrows indicate peri-follicular IFNγ-producing CD4^+^ T cells. Graph shows the location of IFNγ producing CD4^+^ T cells in the skin 30 h following challenge with HSV, *n*=8. Scale bar; 100 μm. **P<0.01, ****P<0.0001, two-tailed paired *t*-test). Data is pooled from two experiments with two to three mice (**a**,**b**) or three experiments with two to three mice (**c**). Flow cytometry plots (**a**,**b**) and images (**c**) are representative of two and three experiments with two to three mice, respectively. Numbers in flow cytometry plots represent frequency of events in the respective gates. Mean and s.e.m (**a**,**c**). D0 and D3; day 0 and day 3 post challenge.

Given the co-localization of APCs and T cells within the peri-follicular clusters, we wanted to determine whether this enhanced the recall response in this vicinity. Firstly, B cell-deficient μMT mice that had resolved infection were challenged with HSV and transferred gDT-II T cells were examined for direct activation by measuring IFNγ production in an *ex vivo* cytokine secretion assay. The skin gDT-II cells were found to rapidly produce IFNγ on secondary infection with HSV, with the highest response between 24 and 48 h after challenge (Fig. 7a). This IFNγ production was extinguished by 72 h (Fig. 7a) and appeared largely confined to skin T cells, since gDT-II cells in the draining lymph were not activated at the time of challenge (Fig. 7b). Importantly, during the peak of the response at 30 h post challenge, the majority of the IFNγ producing skin CD4^+^ T cells were localized to hair follicles, with markedly fewer found in the inter-follicular dermis and epidermis (Fig. 7c). Thus, the hair follicle clusters represent sites of rapid CD4^+^ T-cell activation in skin and are therefore likely to be a major contributor to the secondary response to cutaneous infection with HSV.

## Discussion

Peripheral tissues such as the skin, gut and reproductive tract are exposed to the environment and are therefore susceptible to infection. CD4^+^ T cells are critical in providing protection against secondary infections with HSV^10^,^11^,^12^,^26^. It is therefore important to determine the factors that position these cells at the body surfaces in a way that efficient secondary responses can be mounted. This study shows that a large proportion of all CD4^+^ memory T cells in mice are found in the skin at any given time. This is consistent with what had been reported for the human counterpart^8^. While that earlier study argued that proportionally, T cells in the skin dominated the memory population in humans, with the tissue cells outnumbering those estimated for the circulation, it did not determine what proportion were permanent residents. Here we demonstrate that in mice, the vast majority of skin CD4^+^ T cells at steady state, equilibrate with the circulation rather than remaining permanently fixed in the tissue. Following infection or inflammation, we found that there was a sustained increase in number of locally persisting CD4^+^ T cells. This was due to enhanced recruitment from the circulation, as well as the formation of a longer term resident population. Memory CD4^+^ T cells in previously inflamed skin therefore appeared to be heterogeneous and influenced by the inflammation history of the tissue. Interestingly, at least some of the circulating skin CD4^+^ T cells identified in parabiosis experiments expressed markers usually associated with fixed tissue residency, specifically CD69 and CD103. The latter is consistent with the results from Bromley *et al*.^18^, who directly demonstrated that CD4^+^ memory T cells egressing from the skin express this integrin subunit. Thus, it appears that these molecules may not be useful markers for permanent residency for tissue CD4^+^ memory T cells and that caution should be exercised when using them for this purpose.

Similar to human skin, we found that CD4^+^ memory T cells localize predominantly to the dermis where they preferentially accumulate around skin appendages. The hair follicle has previously been implicated in other important leukocyte functions, most recently in the stress-induced recruitment of dendritic cells into skin^20^. However, even though the follicle isthmus was also implicated in that that study, a key chemokine involved in dendritic cell entry was keratinocyte-produced CCL2 rather than the CCL5 identified here for CD4^+^ memory T-cell recruitment. That the isthmus featured in both forms of leukocyte recruitment may suggest that this region has a wider role to play in skin immune biology. The hair follicle isthmus and bulge are major sites of colonization by commensal microorganisms^27^ and for the production of various important chemokines^20^ and cytokines^28^. Combined, these may act to facilitate the co-aggregation of both T cells and APCs in a highly concentrated fashion. Of note in that respect, professional APCs such as dendritic cells are indeed critical in stimulating localized CD4^+^ T-cell effector activity during active skin infection^24^. Consistent with this, we show that the majority of the skin CD4^+^ T cells that produce the antiviral cytokine IFNγ upon secondary infection with HSV are localized to hair follicles. Thus, peri-follicular clusters may represent sites of preferential T-cell activation in skin, allowing for the induction of rapid secondary immune responses.

Mixed leukocyte clusters have also been implicated in T-cell retention in other tissues, most notably the FRT after HSV infection^12^. Like in skin, those clusters also contained non-T cells, mainly macrophages and dendritic cells. FRT cluster formation was dependent on the chemokine CCL5, similar to what we found for the maintenance of the skin T-cell aggregates. Iijima and Iwasaki^12^ found that CCL5 was produced by CD11b^+^ macrophages with suggestions that they were also involved in ongoing presentation of antigen to the T cells. In the case of hair follicle clusters, there were two important sources of CCL5. These included CD8^+^ T cells, as well as CD11b^+^ cells that most closely resembled a population of monocyte-derived DC^25^. Past studies have shown that CCL5 mRNA is not detected in follicular epidermal cells at least at steady state^20^, consistent with a lack of expression of this chemokine by CD45^−^ cells (data not shown). What attracts these CCL5-producing cells remains unknown at this time, although given the highly localized nature of the clusters, it may be that they are recruited by a subset of hair follicle keratinocytes that produce selective chemokines^20^ that firstly draws in CD8^+^ T cells and monocyte-derived DC, which then secrete CCL5 that subsequently recruits cluster-associated CD4^+^ T cells.

T-cell aggregates have been noted in a number tissues under different circumstances, such as those in mixed leukocyte clusters in the FRT after HSV infection^12^, CD4 T^+^ cells concentrated around lung airways following influenza infection^29^, CD8^+^ T_RM_ cell clusters that remain after vesicular stomatitis virus infection of the brain^30^ and resident CD8^+^ T cells that accumulate in the gut lamina propria after enteric infections with *Yersinia pseudotuberculosis*^13^. Unlike HSV infection of the FRT^12^,^27^, we found that cognate antigen recognition was dispensable for the appearance of T-cell aggregates in the skin as they were found in animals devoid of MHC class II expression. Transient clusters of T cells and DC form around blood vessels 12 h after treatment of skin with DNFB^31^. These peri-vascular effector-type clusters rely on macrophage-derived CXCL2 as opposed to the combined involvement of CCL5 and CD8^+^ T cells for the peri-follicular clusters described here. Although both types of clusters appear to be sites of T-cell activation, the former were found to dissipate after one week. In contrast, peri-follicular clusters remained long-term following the resolution of HSV infection or DNFB treatment, while peri-vascular clusters were rarely seen at these times. The follicular clusters appeared to be critical for ongoing elevation in skin T-cell content, with virtually all the increase in numbers correlating with changes in the peri-follicular T-cell compartment after infection or contact sensitization. Moreover, an increased peri-follicular compartment also reflected an increased recruitment from the circulation, which matched the heightened levels of CCL5 and a number of other chemokines found after infection of the skin.

Both peri- and inter-follicular T cells had similar migration kinetics, despite the latter having a significantly curtailed confinement ratio. Although the majority of clustered cells remained restricted to the area surrounding follicles over the time period examined, a proportion of cells were able to migrate freely in and out of the clusters. Thus, the regions around the follicles were far from static, which is consistent with antigen-recognition being dispensable for the retention of cells in clusters, since T cells have been shown to display a reduction in cell motility on antigen recognition^32^.

Overall, our results reveal a complexity in skin CD4^+^ memory T cells, which preferentially accumulate in clusters localized around the hair follicle isthmus. Such accumulation does not render the T cells static, but instead leaves them in constant movement and undergoing recruitment from the circulation. Importantly, the T-cell content of the skin appears to be altered by inflammation or infection in a way that results in both higher aggregate content and the concomitant increase in recruitment of T cells from the circulation. Although tissue residency has garnered increased attention over recent years, especially for the CD8^+^ T_RM_ subset, our data shows that it should not assumed that all of T cells in peripheral tissues are permanent residents. Given that a proportion of CD4^+^ memory T cells migrate through the skin, in combination with the observation that environmental factors can influence T-cell phenotype^33^,^34^, tissue-specific signals and recirculation has the potential to profoundly influence long-term immunity in peripheral compartments. The extent of this influence and how infection may modify the combination of recirculation, residency and downstream functionality remain critical areas of uncertainty in our overall understanding of immunological memory.

## Methods

### Mice

C57BL/6, B6.SJL-PtpraPep3b/BoyJ (B6.CD45.1), gDT-II × B6.CD45.1, gDT-II × B6.uGFP, B6.uGFP, Kaede, μMT, CCR2^−/−^, CCR8^−/−^ and MHC class-deficient (MHC-II^−/−^) mice were bred and housed in the Department of Microbiology and Immunology at the University of Melbourne, Australia. Female mice between 6 and 12 weeks were used for experiments. The gDT-II mice express transgenic TCRs specific for the HSV-1 glycoprotein-D-derived epitope gD_290–302_. All animal experiments were approved by The University of Melbourne Animal Ethics Committee. C57BL/6 and B6.CD45.1 mice used in parabiosis experiments were purchased from Jackson Laboratories and housed at the animal facility of the Harvard Institute of Medicine, Harvard Medical School. These experiments were performed in accordance with the guidelines set by the Centre for Animal Resources and Comparative Medicine at Harvard Medical School.

### Human subject recruitment

Normal skin samples were obtained as surgical discard from elective breast reduction or abdominoplasty operations (26 donors, age range 18–64). Ethics approval from the relevant institutions was obtained before enrolment of participants in this study and all patients provided written informed consent.

### Viral infection

Skin was infected with the HSV-1 KOS strain by scarification as described^35^. Mice were anaesthetized with ketamine (100 mg kg^−1^) and xylazine (15 mg kg^−1^). Skin was shaved and depilated with Veet then lightly abraded with a dremel, so that the outermost stratum corneum and epidermis was removed. A total of 10^6^ plaque forming units of HSV-1 were applied to the dremel site. Op-site was placed over the virus and mice were bandaged.

### DNFB treatment

A 0.5% solution of DNFB was prepared in a 4:1 mixture of acetone and olive oil. Mice were anaesthetized with ketamine (100 mg kg^−1^) and xylazine (15 mg kg^−1^). Skin was shaved and depilated with Veet before 15 μl of DNFB was applied.

### Parabiotic surgery

Parabiosis surgery was done as described^36^. Briefly, each mouse was anaesthetized with ketamine and xylazine 10 μg g^−1^. Skin was shaved and disinfected by wiping with alcohol prep pads and betadine three times. Matching incisions were made from the olecralon to the knee joint of each mouse and subcutaneous fascia bluntly dissected to create 0.5 cm of free skin. The olecranon and knee joints were attached by a 5-0 silk suture, and dorsal and ventral skin attached by continuous staples or sutures. Betadine was used to cover the entire incision following surgery.

### Adoptive transfer of CD4^+^ T cells

A total of 1 × 10^4^ naive gDT-II cells were isolated from spleen and lymph nodes of gDT-II mice and enriched with magnetic beads^37^, then adoptively transferred via the tail vein. Memory CD4 T cells from CD45.2^+^ HSV memory mice were enriched from spleens and 20–50 × 10^6^ were transferred intravenously into HSV infection-matched CD45.1^+^ memory mice. For polyclonal activated of CD4^+^ T cells, splenocyte and lymph node samples enriched for CD4 T cells were activated by anti-CD3ɛ (5 μg ml^−1^;145-2C11; eBioscience) and anti-CD28 (5 μg ml^−1^; 37.51) *in vitro*. Activated cells (1–2 × 10^6^) were transferred into recipients by intradermal injection (five 20 μl injections over an area of 1.5 × 1 cm^2^ of skin) with a 20-gauge needle. For experiments using pertussis toxin (Sigma-Aldrich), cells were treated for 90 min *in vitro* with 100 ng ml^−1^.

### *In vivo* mouse treatment

For CD4 T-cell depletion experiments, memory DNFB mice were treated with 300 μg of an anti-CD4 antibody (clone GK1.5, Biovest International/National Cell Culture Center) or PBS intravenously on days 0 and 7, as well as 100 μg intra-peritoneally on day 5 of the experiment. For CCL5 neutralization experiments, HSV-1 memory mice were treated intra-peritoneally with 5 μg anti-CCL5 (R & D, 53405) or isotype control antibody for 4 consecutive days, and 1 μg intradermally on day -1. For depletion of CD8^+^ T cells, mice were treated with 100 μg of an anti-CD8 antibody (clone 2.43) for 3 consecutive days before HSV-1 infection, then every 5 days until the time of analysis at day 28–30. For *in vivo* labelling of blood vessel-associated T cells, 3 μg of a phycoerythrin-conjugated anti-CD3 antibody (BD Pharmingen, 145-2C11) was injected intravenously 5 min before the experiment. For recombinant mouse CCL5 (R & D) experiments, 3 μg was injected intradermally into naive mice for 3 consecutive days before analysis. For inhibition of CXCR3, 3 mg kg^−1^ AMG487 (Tocris) was given subcutaneously twice daily for 6 consecutive days.

### Photo-conversion of kaede mice

Mice were anaesthetized with ketamine (100 mg kg^−1^) and xylazine (15 mg kg^−1^). Fur was shaved and skin depilated with Veet. A violet light source (Dymax Bluewave LED 410 nm) was shone from 5 cm away onto a discrete area of skin (∼2 × 2 cm^2^) for 5 min.

### HSV challenge

μMT mice were infected with HSV-1 on the left flank, then challenged with this same virus at least 30 days later. Skin samples were harvested at 24, 48 or 72 h following secondary infection for analysis as indicated.

### Flow cytometry and antibodies

Mice were perfused with 12 ml PBS (Media preparation unit, The University of Melbourne) before skin tissue was harvested into collagenase type 3 (3 mg ml^−1^; Worthington) with DNase (5 μg ml^−1^; Sigma) and chopped into small fragments. Samples were incubated at 37 °C for 90 min then filtered through 75 μm mesh followed by 30 μm mesh. Spleen samples were forced through a wire mesh and filtered through 75 μm mesh. Single-cell suspensions were then stained with antibodies for flow cytometry. The following antibodies from BD Pharmingen were used: fluorescein isothiocyanate-conjugated CD4 (RM4-4, 1/800), CD45.1 (A20, 1/200) and CD11b (M1/70, 1/400), IFNγ (XMG1.2, 1/100). Phycoerythrin-conjugated CD103 (M290, 1/100), IA/IE (M5/114.15.2, 1/200), CD4 (RM4-4, 1/800), CD44 (IM7, 1/200) and CD8 (53-6.7, 1/800). Allophycocyanin-indotricarbocyanine-conjugated TCRβ (H57-597, 1/400) and CD45.2 (104, 1/200). Alexa Fluor-700-conjugated CD4 (RM4-5, 1/200) CD45.2 (104, 1/200). Purified anti CD16/32 was used to block nonspecific Fc receptor binding (2.4G2, 1/200). The following antibodies from eBioscience were used: fluorescein isothiocyanate-conjugated CD90.2 (30-H12, 1/3000). Phycoerythrin-conjugated Foxp3 (FJK-16 s, 1/100). Phycoerythrin-indotricarbocyanine-conjugated CD45.1 (A20, 1/200), CD11c (N418, 1/400) and rat IgG2b kappa iso control (eb149/10H5, 1/00). Allophycocyanin-conjugated CD69 (H1.2F3, 1/200), CD45.2 (104, 1/200), F4/80 (BM8, 1/100). Alexa Fluor-700-conjugated IA/IE (M5/114.15.2, 1/200). The following antibodies from Biolegend were used: Phycoerythrin-conjugated CD64 (X54-5/7.1.1, 1/100) and CCL5 (2E9/CCL5, 1/100). Allophycocyanin-conjugated CD103 (2E7, 1/200). Propidium iodide or a live/dead stain (Invitrogen) was used to exclude dead cells. SPHERO calibration particles (BD pharmingen) were added to samples to allow calculation of cell numbers. A FACSCanto II (BD) and Flowjo software (TreeStar) were used for analysis.

### *Ex vivo* intracellular cytokine staining

For IFNγ production following HSV challenge, 0.25 mg ml^−1^ brefaldin A (Sigma) was injected intravenously 6 h before harvest. Skin samples were then incubated with 10 μg ml^−1^ brefeldin A in collagenase for 90 min at 37 °C. Intracellular staining was performed according to the manufacturers instruction (eBioscience Foxp3 staining kit). Briefly, after washing the surface stain, cells were resuspended in Fix/Perm buffer and incubated on ice for 30 min. Cells were washed and resuspended in 100 μl perm/wash buffer. Two microlitre of both normal rat serum (eBioscience) and normal mouse serum (eBioscience) was then added to each sample and incubated at room temperature (RT) for 15 min. Intracellular antibodies were directly added and incubated for a further 45 min at RT.

### Immunofluorescent histology

Skin was harvested into 20% sucrose (Sigma-Aldrich) in PBS for 30 min on ice and snap frozen in tissue-tek OCT compound (Sakura Finetek) with liquid nitrogen. If GFP^+^ cells were present, skin was harvested into periodate-lysine-paraformaldehyde (PLP) buffer (0.2 M NaH_2_PO_4_, 0.2 M Na_2_HPO_4_, 0.2 M L-lysine and 0.1 M sodium periodate with 4% paraformaldehyde) for 30 min, washed twice in PBS then incubated in PBS with 20% sucrose for 30 min on ice. Frozen samples were cut into 20 μm thin slices using a microtome (Leica) and placed on charged glass slides. Samples were air-dried for at least 2 h then rehydrated with PBS for 5 min. Protein block (DAKO) was applied for 30 min RT in a semi-humidified chamber. Sections were stained with Alexafluor-conjugated antibodies for 30 min at RT then washed four times for 3 min with PBS. If purified antibodies were used, they were incubated for 90 min at RT following protein block, washed with PBS and an appropriate anti-species antibody was then incubated for 30 min at RT. Hoechst 33342 (Biorad) was added for 3 min to visualize cell nuclei and then washed in PBS before mounting.

For *ex vivo* staining of IFNγ by histology, 0.25 mg ml^−1^ brefaldin A was injected intravenously 6 h before harvest. Skin was fixed in PLP, then cut and stained as usual. The following antibodies from BD were used Alexa Fluor-647-conjugated IFNγ (XMG1.2, 1/100) and Alexa Fluor-647-conjugated IgG1 isotype control.

The following antibodies from Biolegend were used: Alexa Fluor-488-conjugated CD8α (53–6.7, 1/100), Alexa Fluor-488-conjugated CD4 (GK1.5, 1/100) and Alexa Fluor-488-conjugated CD11c (N418, 1/100); Alexa Fluor-647-conjugated CD4 (RM4-5, 1/100), Alexa Fluor-647-conjugated CD11c (N418, 1/100) and Alexa Fluor-647-conjugated TCRβ (H57-597, 1/100). Purified IA/IE (M5/114.15.2, 1/400) and polyclonal HSV (DAKO, 1/30) were detected by an appropriate secondary anti-species antibody conjugated to Alexa-Fluor 568 (Life Technologies, 1/800). The following purified antibodies used to stain human skin samples were used: CD3 (clone CD3-12, AbD Serotec, 1/200), CD4 (clone YNB46.1.8, AbD Serotec, 1/100) and CD11c (clone B-Ly6, BD, 1/100). These antibodies were detected with appropriate secondary anti-species antibodies conjugated to an Alexa-Fluor (Life Technologies, 1/800).

### Confocal microscopy and imaging analysis

Tiled (3–5 × 1), *z*-stacked images were acquired with a Zeiss LSM700 confocal microscope at × 20 zoom and processed with Imaris 8 (Bitplane) software. Cells in images were counted manually (15–100 single images per mouse) with the cell counter plugin in ImageJ64 software, or if a cluster was too dense to distinguish single cells, fluorescence intensity was used. For this, the average fluorescence intensity of 5–10 single cells in every image was determined then the number of cells in a cluster was extrapolated from that value. Non-serial sections (100 μm between sections) were counted to obtain an accurate representation of the skin. The number of cells per cm^2^ skin was determined by extrapolating from the number in a 20 μm section. A correction factor was applied to exclude cells that span two sections that would have been counted twice^38^.

### Intravital 2-photon microscopy

Skin flank from mice was imaged as described previously^39^. Briefly, mice were anaesthetized using a mixture of isoflurane (Cenvet), air and oxygen. Two small parallel incisions were made on the shaved and depilated area of skin flank to be imaged. Skin flank was adhered onto a stainless steel plate, secured on a custom-build stage and stabilized under a #1 glass coverslip. Mice were imaged using a Zeiss 710 NLO Axio Observer microscope (Zeiss) equipped with a Coherent Chameleon Vision Ti:sapphire laser tuned to 920 nm. Hair follicle clusters were imaged at depths between 40–100 μm below the skin surface for periods of 45 min to 1 h. Time-lapse *Z*-stacks were acquired at 60 s intervals, and analysed using Imaris 8.

### Intravital imaging analysis

To segregate inter-follicular cells from peri-follicular cells, tracks were generated using Imaris Spot function, and hair follicles were rendered as a three-dimensional surface. Tracks were computed using a custom-modified version of the script ‘Surface to Spot Distance' through a Matlab interface (Version 14; MA. USA). To calculate the T-cell dynamics around or between the clusters, we determined the centre of each cluster and defined the gDT-II T cells remaining within a 50 μm distance from this point (defined by the average cluster diameter) for more than 10 min as belonging to a cluster, while the remainder were determined to be non-cluster. Statistical values for mean track speed and confinement were extracted from Imaris software. Note: the confinement index was calculated based on the Track Straightness value in the Imaris Track statistical output. Track straightness, defined as (Track Displacement Length/Track length), denotes the likelihood that a cell migrates in a straight line. Here we show confinement index, the opposite of Track Straightness (1-[Track Straightness]), as a measure of the likelihood that a cell migrates within a confined path.

### Quantitative RT–PCR

For quantitative PCR with reverse transcription (RT–PCR) analysis of chemokine expression, HSV-1 memory mice were perfused and areas of skin (0.5 × 0.5 cm^2^) from previously infected and control flanks were harvested in RNA later (Ambion). RNA from whole skin was extracted with a Fibrous tissue kit (Qiagen) according to the manufacturer's instructions. cDNA was synthesized with SuperScript III Reverse Transcriptase (Invitrogen) and oligo(dT) primers (Promega). Taqman fast Advanced Mastermix (Life Technologies), Taqman gene expression assays and a StepOnePlus Real-Time PCR system (all Life Technologies) were used for quantitative RT–PCR experiments. The cycling threshold (Ct) of gene transcripts for each skin sample was determined by RT–PCR and normalized to the geometric mean of the Ct values of housekeeping genes *GAPDH*, *β2m* and *TBP* for calculation of the Δ*Ct* value. The expression of chemokines was determined by comparing the Δ*Ct* of each previously infected skin sample with the Δ*Ct* of the corresponding control skin from each mouse using the 2^−ΔΔCt^ method^40^. The following Taqman gene assays (Life Technologies) were used: Cxcl4; Mm00451315_g1, *Cxcl9*; Mm00434946_m1, *Cxcl10*; Mm00445235_m1, *Cxcl14*; Mm00444699_m1, *Cxcl16*; Mm00469712_m1, *Ccl1*; Mm00441236_m1, Ccl2; Mm00441242_M1, *Ccl5*; Mm01302427_m1, *Ccl6*; Mm01302419_m1, *Ccl8*; Mm01297183_m1, *Ccl9*; Mm00441260_m1, *Ccl19*; Mm00839967_g1, *Ccl20*; Mm01268754_m1, *Ccl21*; Mm03646971_gH, *Ccl27*; Mm04206819_gH, *Gapdh*; Mm99999915_g1, *Tbp*; Mm00446973_m1, *β2m*; Mm00437762_m1.

### Data and statistical analysis

Data was plotted and statistics determined using prism (Graphpad) software. *P* values were determined by an appropriate test (noted in figure legends) with differences between groups considered significant for *P* values of <0.05.

### Data availability

We declare that the data supporting the findings of this study are available within the article and its Supplementary Information files.

## Additional information

**How to cite this article:** Collins, N. *et al*. Skin CD4^+^ memory T cells exhibit combined cluster-mediated retention and equilibration with the circulation. *Nat. Commun.* 7:11514 doi: 10.1038/ncomms11514 (2016).

## Supplementary Material

Supplementary Information Supplementary Figures 1-8

Supplementary Movie 1 Migration pattern of perifollicular CD4+ T cells. 104 naïve gDT-II GFP cells were transferred to B6 mice, which were infected the following day and imaged at the indicated time points. Example intravital imaging of clustered gDT-II cells (green) around hair follicles (auto-fluorescent hair in green) at memory (30-70 days) post infection with HSV-1. The dermis is identifiable by collagen (second harmonic generation, blue). Representative of 5 movies from 5 mice.

Supplementary Movie 2 Analysis of the migration pattern of perifollicular CD4+ T cells. Example intravital imaging of clustered gDT-II cells (green) around hair follicles (auto-fluorescent hair in green) at memory (31 days) post infection with HSV-1. The dermis is identifiable by collagen (second harmonic generation, blue). gDT-II cell migration was analysed (white tracks), then cells were split into peri-follicular (magenta) and follicular compartments (turquoise) for further analysis. Representative of 5 movies from 5 mice.
