# Supplementary material for: Skin CD4+ memory T cells exhibit combined cluster-mediated retention and equilibration with the circulation
Source: Nat Commun. 2016 May 10;7:11514. doi: 10.1038/ncomms11514 (PMC4866325; doi:10.1038/ncomms11514)
Supplement: Supplementary Information — Supplementary Figures 1-8 [file ncomms11514-s1.pdf]

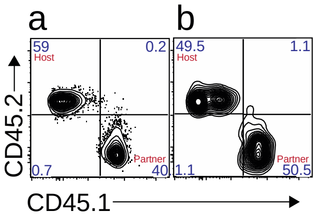

Supplementary Figure 1. Skin CD4+ T cells are in equilibrium with the circulation. Representative flow cytometry plots of spleen (**a**) and skin (**b**) CD4 T cells of the CD45.2+ mouse in parabiosis experiments of (**Fig. 1d**, **e**) with naïve mice, as indicated. Representative of 3 experiments with 4–5 parabiotic pairs. Gated on CD4+ cells, numbers in plots represent frequency of events in the respective gates.

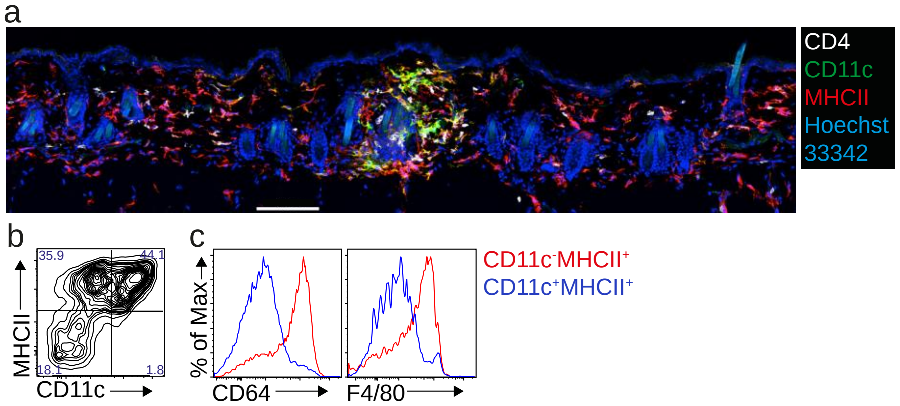

Supplementary Figure 2. APC cluster around hair follicles. (**a**) Naïve flank skin, detecting perifollicular CD4 (white), CD11c (green) and MHC II (red) positive cells. Scale bar, 100 µm. (**b**) CD11c and MHC class II expression by antigen presenting cells. Gated on CD45.2+ live cells. (**c**) CD64 and F4/80 expression by CD11c– MHC II+ (red) and CD11c+ MHC II+ (blue) cells in (**b**). Image and plots representative of 3 similar experiments with 3–5 mice. Numbers in plot of (**b**) represent frequency of events in the respective gates.

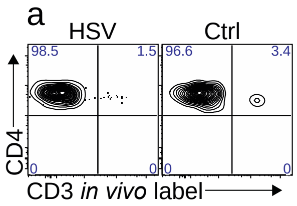

Supplementary Figure 3. $CD4^+$ T cells are largely contained within the skin parenchyma**.** (**a**) HSV memory mice were injected intra-venously with an anti-CD3 PE antibody 5 min prior to analysis. Representative plots of memory skin on the infected (left) and control sides (right), gated on CD4 T cells. Numbers in plots represent frequency. Data representative of 2 experiments with 5 mice.

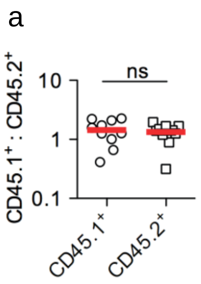

Supplementary Figure 4. Splenic $CD4^+$ T cells are in equilibrium following parabiosis**.** (**a**) $CD45.1^+$ mice were treated with DNFB 3 wks before being surgically joined to naïve $CD45.2^+$ mice for 8 wks. Graph shows the ratio of host and partner CD4 T cells in spleens of both mice of each pair. Symbols represent individual mice. NS, not significant (two-tailed paired t-test). Pooled data from 2 experiments with 5 pairs of parabiotic mice in each. Mean.

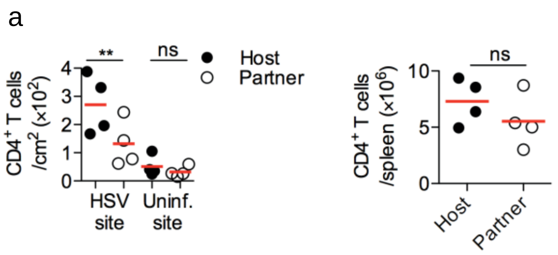

Supplementary Figure 5. Memory CD4+ T cells in previously infected skin appear heterogeneous. CD45.1+ and CD45.2+ mice were infected with HSV 124 days before being joined by parabiotic surgery. Mice were left joined for 7 wks before analysis. Skin was harvested from the HSV site and uninfected skin near the base of the tail from each mouse. Graphs show the number of host and partner derived CD4 T cells in the skin (HSV and uninfected sites) and spleen. Two pairs of mice are shown. Symbols represent individual mice. NS, not significant; ** $P < 0.01$ (two-tailed paired t-test). Mean. Uninf.; uninfected.

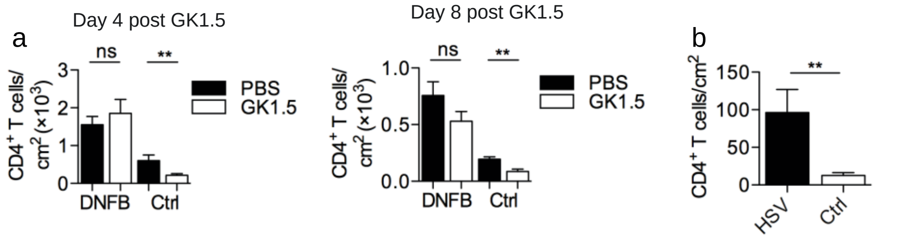

Supplementary Figure 6. Previously inflamed skin recruits and retains memory CD4^{+} T cells. (**a**) Absolute number of skin CD4^{+} T cells from **Fig. 3I**, in which DNFB memory (28-70 days following treatment) mice were treated with an anti-CD4 depleting antibody, *n* = 20 per group at d 4 and 11 at d8 from 4 and 2 experiments respectively. (**b**) Absolute number of transferred CD4^{+} T cells from **Fig. 3J**, in which splenic CD4^{+} T cells from CD45.2^{+} HSV-1 memory mice (33–63 days following infection) were transferred intra-venously into infection matched CD45.1^{+} mice, *n* = 15 from 5 experiments. NS, not significant; ** $P < 0.01$ (two-tailed Mann-Whitney test). Mean and s.e.m.

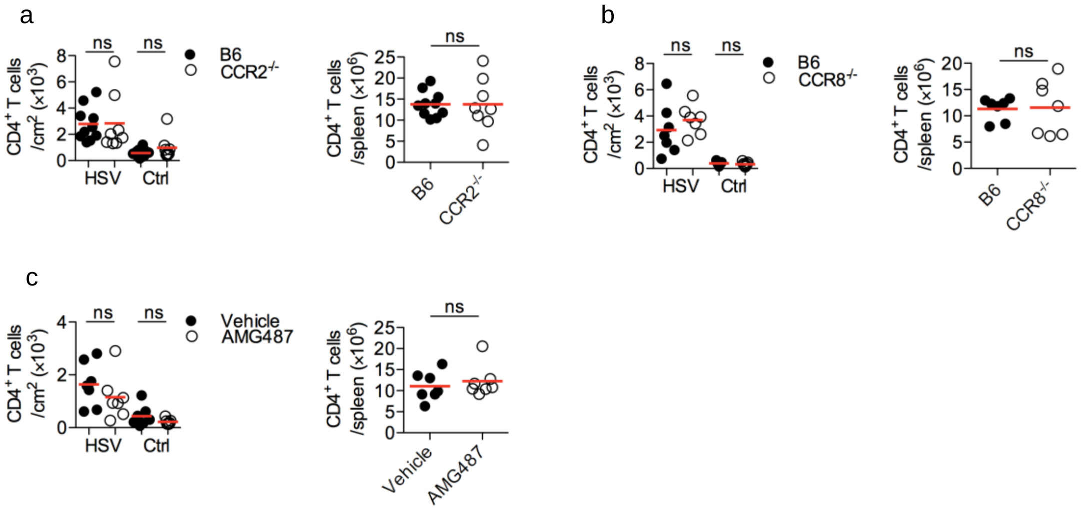

Supplementary Figure 7. Skin CD4 T cell increase is not regulated by CCL1, CCL2, CCL8, CXCL9 or CXCL10. (**a**, **b**) Number of $CD4^+$ T cells in previously HSV infected (34–80 days following infection) and control skin, as well as spleen, of $CCR2^{-/-}$ (**a**), $CCR8^{-/-}$ mice (**b**) compared to WT controls. (**c**) Number of $CD4^+$ T cells in previously HSV infected (44–48 days following infection) and control skin, as well as spleen, following AMG487 (CXCR3 inhibitor) treatment compared to vehicle treated control mice. Data pooled from 2 experiments. Symbols represent individual mice. NS, not significant (two-tailed Mann-Whitney test). Mean.

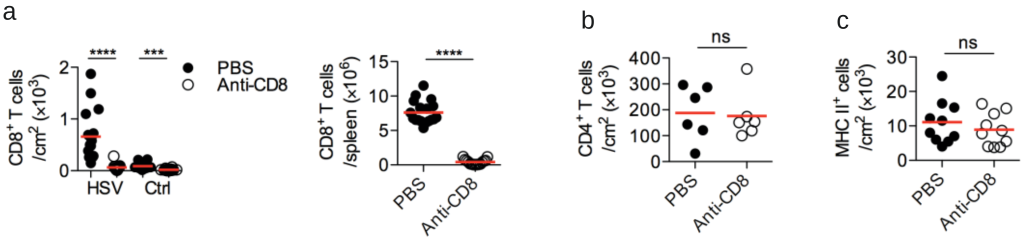

Supplementary Figure 8. Prolonged CD8+ T cell depletion is efficient. (**a**) The number of CD8+ T cells in skin (previously infected and control) and spleen in experiments of **Fig. 5G**, in which mice were depleted of CD8+ T cells prior to HSV infection, then every 5 days until analysis at d 28–30. (**b**) Number of CD4+ T cells in skin at d 8 following HSV infection in the absence of CD8+ T cells. (**c**) Number of MHC II+ cells at d 28–30 following HSV infection in the absence of CD8+ T cells. Symbols represent individual mice. Data pooled from 4 (**a**) or 2 (**b**, **c**) experiments. NS, not significant; *** $P < 0.001$, **** $P < 0.0001$. (two-tailed Mann-Whitney test). Mean.
